# Teachers’ Heart Rate Variability and Behavioral Reactions in Aggressive Interactions: Teachers Can Downregulate Their Physiological Arousal, and Progesterone Favors Social Integrative Teacher Responses

**DOI:** 10.3390/ejihpe14080149

**Published:** 2024-08-03

**Authors:** Alexander Wettstein, Sonja Krähling, Gabriel Jenni, Ida Schneider, Fabienne Kühne, Martin grosse Holtforth, Roberto La Marca

**Affiliations:** 1 Department of Research and Development, University of Teacher Education Bern, Fabrikstrasse 8, 3012 Bern, Switzerlandgabriel.jenni@phbern.ch (G.J.); fabienne.kuehne@phbern.ch (F.K.); roberto.lamarca@clinica-holistica.ch (R.L.M.); 2 Clinical Psychology and Psychotherapy, Department of Psychology, University of Bern, Fabrikstrasse 8, 3012 Bern, Switzerland; martin.grosse@unibe.ch; 3 Psychosomatic Medicine, Department of Neurology, Inselspital, Bern University Hospital, Haus C.L.Lory, 3010 Bern, Switzerland; 4 Centre for Stress-Related Disorders, Clinica Holistica Engiadina, Plaz 40, 7542 Susch, Switzerland; 5 Department of Clinical Psychology and Psychotherapy, University of Zurich, Binzmühlestrasse 47, 8050 Zurich, Switzerland

**Keywords:** teacher stress, student aggression, teacher reaction, state space grids, heart rate variability, polyvagal theory, progesterone, psychological strain

## Abstract

Aggressive student behavior is considered one of the main risk factors for teacher stress. The present study investigated teachers’ physiological and behavioral reactions when facing aggressive student behavior and examined which resources favor adaptive teacher reactions. The sample included 42 teachers. We assessed (a) teacher self-reports (i.e., resources, risk factors, and vital exhaustion) (b) classroom observations, (c) ambulatory assessments of teachers’ heart rate and heart rate variability, and (d) teachers’ progesterone concentrations in the hair. The present study focused on a subsample of ten teachers (9 females, *M*age = 34.70, *SD* = 11.32) managing classes which were potentially very stressful as they had a high density of aggressive behavior. High levels of work satisfaction, hair progesterone, and a low level of work overload fostered social integrative teacher responses. Moreover, in 75% of the cases, teachers succeeded in downregulating their physiological reaction. Our results support the notion that teachers evaluate stressors in light of their resources. When they perceive their resources as insufficient for coping with a challenging situation, stress arises, and subsequently, they react inefficiently to aggressive behavior. Thus, teacher education could benefit from strengthening teacher resources and strategies for coping with aggressive student behavior.

## 1. Introduction

### 1.1. Student Aggression as a Central Source of Teacher Stress

Compared to other professions, teachers report higher levels of self-perceived workplace stress [1,2] and rates of burnout [3]. This seems to be partially due to characteristics of the teaching profession such as a high density of demanding interactions [4]. Teachers face unstructured problems, dynamic situations, and multiple demands (e.g., unpredictable social interactions or complex challenges with no clear solution such as student motivational problems, misbehavior, or individual learning needs) [5]. At the same time, they act under external pressure, such as exaggerated social expectations and pressure from parents and school management. They have to enforce rules and have to manage the class as a whole while trying to monitor and support each individual as best as possible [6,7]. One especially outstanding stressor is aggressive student behavior [8,9,10,11]. Aggression is any verbal or physical behavior directed toward another individual with the proximate (immediate) intent to cause harm [12]. Aggressive student behavior has many faces (e.g., hitting, insulting, or mocking others). However, student aggression is not an isolated event with a single actor but rather a complex process involving students, teachers, their interactions, as well as their individual stress experiences.

Generally, teachers’ reactions to aggressive student behavior in three overarching categories: punitive (threatening, punishing, and belittling), neutral (observing/ignoring, stopping, and admonishing), and social integrative (suggesting a compromise, integrating, encouraging, empathizing, and using humor) [13,14]. While social integrative responses are considered favorable, neutral or punitive measures are deemed ineffective in preventing aggressive student behavior. Observational studies support this notion and show that teachers oftentimes ignore or punish aggressive behavior and may thereby even trigger further disruptions [14].

Therefore, understanding episodes of aggression in the classroom as interactive events is crucial. The state space grids (SSG) method assesses interactional episodes [15,16] and analyzes interactions over time. In SGG, the behavior of one party (e.g., aggressive student behavior) is represented on the x-axis and that of the other party is represented on the y-axis (e.g., a teacher’s reaction to aggressive student behavior). Combining the x- and y-axis results in a holistic snapshot of the interaction system (e.g., aggression/ignoring). With each interactional turn, defined by the change of actor, the behavior of the interactional parties and the resulting state of the interactional system are re-assessed. Through this, the SSG method allows for a microanalysis of aggressive interactions between students and teachers over time.

The SSG method has initially been applied to study parent–child interactions [17,18,19] and later also to teacher–student interactions [20,21,22,23,24,25,26]. These studies show that in challenging interactional situations, there is a risk of developing rigid and escalating interactional patterns (e.g., a cycle of aggressive student behavior, punitive teacher behavior, and increased student aggression). Thus, by using inappropriate responses to aggressive student behavior (e.g., by ignoring or punishing), teachers may unintentionally reinforce the problems they originally tried to solve. Teacher stress may aggravate dysfunctional interactions, resulting in vicious cycles in which stressed teachers react unfavorably to student aggression, leading to even more teacher stress, followed by more aggressive student behavior, and so on. To our knowledge, however, studies to date have not assessed interactions of aggressive student behavior, teachers’ subsequent reactions, and their experiences of psychological and physiological stress using microanalytic methods.

### 1.2. Risk Factors and Resources

Teachers’ risk factors and resources might influence their responses to aggressive student behavior. Stressful job characteristics place considerable demands on teachers. Following Lazarus and Folkman’s [27] stress model, teachers evaluate potential stressors on the basis of the perceived availability of resources for coping with these challenges. The following presents a selection of potential risk factors and resources for teacher stress and health.

Possible risk factors for stress in the teaching profession include pressure to succeed, work overload, occupational problems, self-related problems, and vital exhaustion. The teaching profession is characterized by fuzzy boundaries (i.e., one can always do more and try to improve). Accordingly, an excessive readiness to spend time and energy and to strive for perfection are risk factors for teachers to experience high levels of stress.

Teachers may also experience external pressure from school administration, other teachers, parents, and students. When teachers are constrained by the curriculum and held accountable for student performance or when they perceive students as unmotivated, they may become pressured and are more likely to control students [28,29,30]. Pressure on teachers predicts lower intrinsic motivation for teaching [29,31]. Furthermore, teachers’ intrinsic motivation is negatively related to teachers’ perception of classroom overload and students’ disruptive behavior [32]. Accordingly, job pressures are positively associated with burnout and work dissatisfaction in teachers [31,32,33,34]. Thus, when teachers experience less motivation because of job pressures, they are more likely to be more controlling with students, and the risk of maladaptive teacher responses to aggressive student behavior increases.

Empirically supported possible resources ameliorating stress in the teaching profession include positive experiences, high work satisfaction [35], support from the school team and school administration [36], as well as high levels of the hormone progesterone [37]. A personal ability to set limits and temporarily disengage from stressors might also be a protective factor in the teaching profession. It is, therefore, important that teachers take time to engage in such activities that “recharge their batteries”.

Work is assumed to be a central part of most people’s identities [35,38]. In this study, work satisfaction refers to experiencing one’s job not only as a financial necessity but also as a meaningful part of one’s identity and an opportunity to feel socially connected and appreciated. Accordingly, work satisfaction is strongly and consistently related to subjective well-being [38].

To successfully master challenging social interactions in everyday school life, teachers depend on functioning cooperations in multi-professional teams. For many mammals, particularly primates, seeking social support is an important part of coping with stress, and research in multiple fields has documented positive or protective health effects of social support for humans [39]. In the teaching realm, a positive social school climate, including social support as well as positive relations with colleagues, parents, and students, seems to protect teachers from burnout [40,41,42,43].

In addition to self-reported psychological variables, physiological markers such as hormones relate to teachers’ coping abilities. Beyond its reproductive function, the hormone progesterone has been found to be associated with the motivation to bond with other humans [44] and seems to have a stress-buffering effect [39]. Relatedly, experimental studies have shown links between progesterone and increased affiliation motivation [45] and lower levels of aggression [46]. A further experimental study showed that administering high dosages of progesterone in men dampened acute psychological stress responses [37]. However, above and beyond these psychological findings, the effects of progesterone on physiological stress responses are far less clear.

In sum, we assume that psychological and physiological resources (e.g., high work satisfaction, the tendency to seek positive experiences, social support, and high progesterone) foster social integrative (and thereby adaptive) teacher responses to student aggression. In contrast, risk factors (e.g., pressure to succeed) might be associated with teacher stress and maladaptive teacher responses, such as ignoring or punishing students.

### 1.3. Physiological Teacher Stress in the Light of Polyvagal Theory

Aggressive interactional episodes challenge teachers. It can be assumed that the associated stress manifests not only in the teacher’s psychological experience but also in the teacher’s physiological reactions. Heart rate (HR) and heart rate variability (HRV) are reliable indicators of physiological stress [47]. HR reflects the number of heart contractions (beats) per minute [48]. HRV represents the variance in the time intervals between successive heartbeats. Stressful situations usually increase HR and decrease HRV [47].

However, only a few researchers have examined the cardiovascular activity of teachers in daily life [49]. Two studies found significantly higher HRs in teachers on teaching days than on leisure days [50,51]. Accordingly, one study found lower HRV in teachers on workdays compared to leisure days [52]. Donker and colleagues [53] investigated how teachers’ moment-to-moment interpersonal behavior is associated with their HR. They found that high levels of observed teacher agency (e.g., active leading behavior) went together with a high HR. However, it is unclear how teachers’ HR and HRV are associated with aggressive student behavior.

**Figure 1:**
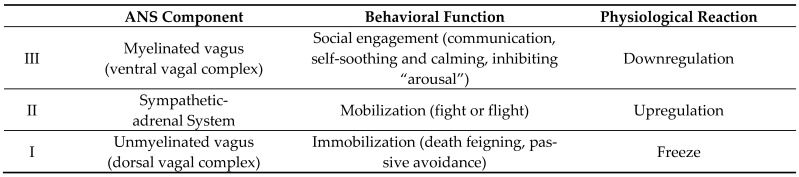
Three autonomic nervous system (ANS) components and their behavioral functions.

The polyvagal theory [54,55] is a phylogenetic model that specifies the association between the evolution of the autonomic nervous system (ANS, consisting of the sympathetic and parasympathetic branch) and social communication (e.g., affective experiences, emotional expression, vocal communication, facial gestures, and social behavior). Communication is central to the teaching profession, especially in aggressive interactions. The polyvagal theory assumes that the cranial vagal nerve, which is central to the parasympathetic nervous system, is further structured into a ventral vagal system and a dorsal vagal system. Thus, the theory distinguishes three ANS components with three corresponding behavioral functions (see Figure 1).

Downregulation (social engagement). The ventral vagus is myelinated, which enables rapid transmission of nerve signals and, thus, highly dynamic adaptation to the environment [54]. It inhibits sympathetically controlled fight or flight behavior, regulates the HR, and acts as a vagal brake, preventing the sympathetic nervous system from influencing the heart’s activity [54]. Central to activating the social engagement system is the perception of safety about the internal or external situation [56].

Upregulation (mobilization; fight or flight). If one does not succeed in resolving a challenge through social interaction or if a situation is subjectively perceived as physically or socially threatening, the sympathetic nervous system is assumed to be activated [56]. The sympathetic nervous system is the second oldest part of the ANS [57] and prepares the organism for the fight or flight response [57]. Physiologically, its activity is associated with an increase in heart rate, blood pressure and respirational rate, blood redistribution, and muscle tightening to provide the body with the energy and oxygen needed to fuel a rapid response to danger [57]. However, this mode reduces effective social communication and the application of interactional skills [57]. Instead, the behavior reflects fight (emotion of anger) or flight (emotion of fear) with the quickening of thoughts and scanning attention for potential sources of threat [57].

Freeze (immobilization). If mobilization is insufficient for dealing with the stressor, the dorsal vagal complex is assumed to come into play [56]. The dorsal vagal complex manifests in immobilization behavior (e.g., decreasing HR, decline of bloody oxygen, sense of dread, and solidification of the body) and may be perceived as “playing dead”. However, freezing behavior occurs only in extremely stressful situations going far beyond a threat a teacher might perceive when facing common student aggression.

Thus, physiologically, teachers can react to aggressive student behavior with either down- or upregulation. However, teachers’ upregulation might be associated with elevated psychological strain in the long run. We distinguish different concepts of psychological strain. Work overload is a potential consequence of ongoing occupational stress [58] when job demands exceed an individual’s potential to deal with it. Occupational problems are associated with negative feelings related to work. Self-related problems include low frustration tolerance, feelings of depersonalization, or concentration problems. Vital exhaustion is a psychosomatic state of unusual fatigue, lack of energy, irritability, and demoralization [59]. Stress is also positively associated with the body mass index (BMI; kg/m^2^) and adiposity [60,61]. For example, Harding et al. [62] found that psychosocial stress (perceived stress and stressful life events) led to weight gain over five years.

Finally, teachers’ psychological strain may partially explain their responses to aggressive student behavior. Prior stress might impair teachers’ ability to cope effectively [36,63] and might affect teachers’ reactions when facing student aggression, further reinforcing the vicious cycle.

### 1.4. Present Study

This study is part of a larger ambulatory assessment project on stressful classroom interactions [36,64,65]. The present exploratory study investigated teachers’ behavioral and physiological reactions when facing aggressive student behavior and examined which resources foster adaptive teacher reactions. Previous research relied primarily on questionnaires, focused on individual behavior, and neglected physiological as well as interactional processes. Our ambulatory assessment study [65] not only relies on questionnaires but also captures the processes occurring in the classroom with systematic behavioral observation, microanalytically examines the emerging interactions between students and teachers, examines the association between teachers’ risk factors and resources and the teachers’ responses to aggressive student behavior, and takes physiological processes into account.

### 1.5. Research Questions and Hypotheses

**Hypothesis** **1.** *Which aggressive student behaviors occur, and how do teachers respond? Based on previous research, we expect that teachers mostly ignore aggressive student behavior.*

**Hypothesis** **2.** *Which interactional episodes develop? We expect teachers to respond mostly neutrally to overall student behavior, and the most common combination is aggressive student behavior and ignoring teacher behavior.*

**Hypothesis** **3.** *Which factors foster adaptive teacher responses to aggressive student behavior? We expect that high levels of teacher resources foster social integrative teacher reactions, whereas risk factors are associated with punitive teacher reactions.*

**Hypothesis** **4.** *How do teachers physiologically respond to aggressive student behavior? Following Porges’s polyvagal theory [54,55], teachers can react with social engagement (downregulation) or mobilization (upregulation). Based on the current state of research, we examine this question exploratively. However, we expect high levels of teacher resources to be positively associated with downregulation, while we expect strong risks and high psychological strain to be associated more strongly with mobilization (Hypothesis 4).*

**Hypothesis** **5.** *How are up- and downregulation associated with teachers’ psychological strain longitudinally? We expect that downregulation will be associated with lower levels of teachers’ psychological strain, while mobilization will increase it in the long term.*

## 2. Methods

### 2.1. Participants

The original study included 42 teachers (28 females, *M*age = 39.66, *SD* = 11.99). Participants were recruited via flyers and circular emails. The inclusion criteria for participation in this study were employment as a primary or secondary teacher in the canton of Bern and a workload of at least 16 lessons per week (equivalent to at least 60 percent employment). Exclusion criteria were working outside of the canton of Bern, acute infections, cardiovascular or other chronic diseases, usage of cardiovascular drugs or other medication in the past two months (except phytopharmaceuticals), substance abuse, consumption of psychoactive substances in the last four weeks, more than two standard alcoholic drinks per day, smoking more than ten cigarettes per day, long-distance flights within the last two weeks, as well as pregnancy. All teachers were screened for inclusion and exclusion criteria in a short interview. The enrolled participants signed informed consent. This study was approved by the ethics committee of the canton of Bern and by the Internal Review Board (IRB) of the University of Bern. It was conducted in strict compliance with current data protection laws and in accordance with the declaration of Helsinki.

In this study, we used complex microanalytical methods to examine a subsample of 10 classes with the highest intensity of aggressive student behavior. The teachers of this subsample were similar to the overall sample of teachers. However, they teach so-called hot-spot classes (i.e., classes in which aggressive student behavior occurs more frequently). We decided that a minimum threshold value of aggressive interactional sequences must be reached for the phenomena to become sufficiently clear to be investigated, such as the developing interactional patterns and HRV effects. The subsample for the present microanalysis included 10 teachers (9 females, *M*age = 34.70, *SD* = 11.32). In total, 19 interactional episodes containing aggression with a total time of 21.95 min were subjected to microanalysis. During this time, 258 student behaviors and 150 teacher responses met our inclusion criteria.

### 2.2. Measures

In the present study, measurements included (a) self-reports (i.e., questionnaires assessing teachers’ resources, risk factors, and psychological strain), (b) classroom observations (i.e., 19 interactional episodes with a high density of aggressive behaviors), (c) ambulatory assessment of teachers’ HR and HRV, and (d) teachers’ BMI and progesterone concentration in the hair. The longitudinal assessment of different psychological strain measures (i.e., work overload, occupational problems, self-related problems, vital exhaustion, and BMI) included three waves: baseline (t0), 1-year follow-up (t1), and 2-year follow-up (t2).

#### 2.2.1. Self-Reports

Teachers provided information on demographic variables and completed the following questionnaires:

Seeking positive experiences was assessed by using a self-developed scale with two items (“I make time to do things that are good for me”. “I often don’t have time for my favorite activities”. A 5-point Likert scale ranging from 1 (strongly disagree) to 5 (strongly agree) was used. The Spearman–Brown coefficient was *r_tt_* = 0.75).

Perceived social support from other teachers and school administration was assessed by using a self-developed scale with two items. Support from other teachers was measured with the item “When I need advice, other teachers are there for me”. Support from school administration was assessed with the item “When I feel overwhelmed, I am supported by the school administration”. A 5-point Likert scale ranging from 1 (strongly disagree) to 5 (strongly agree) was used. Because the correlation between the two items was moderate, with a Spearman–Brown coefficient of *r_tt_* = 0.54, we analyzed them separately as two single-item measures.

Work satisfaction was assessed with a subscale of the Burnout Screening Scales (BOSS III) [66]. Five items were rated from 1 (does not apply) to 6 (applies strongly). The scale captures identification with the profession (“What I do at work is an important part of me.”) and professional social relatedness (“I feel connected to my colleagues and school.”). Reliability was α = 0.65.

Pressure to succeed was assessed with a subscale of the Trier Inventory of Chronic Stress (TICS) [67]. Nine items were rated from 1 (never) to 5 (very often). A sample item is “Situations in which it depends entirely on me whether a contact with another person is satisfactory”. Reliability was α = 0.82.

Work overload was assessed with a subscale of the Trier Inventory of Chronic Stress (TICS) [67]. Eight items were rated from 1 (never) to 5 (very often). A sample item is “I have too little time to fulfill my daily tasks”. Reliability was α = 0.95.

Occupational problems were assessed with a subscale of the Burnout Screening Scales (BOSS I) [66]. Ten items were rated from 1 (does not apply) to 6 (applies strongly). A sample item is “I have lost the joy of my work”. Reliability was α = 0.91.

Self-related problems were assessed with a subscale of the Burnout Screening Scales (BOSS I) [66]. Ten items were rated from 1 (does not apply) to 6 (applies strongly). A sample item is “I feel that even small demands are a burden”. Reliability was α = 0.91.

Vital exhaustion was assessed with the German translation [68] of the Maastricht Vital Exhaustion Questionnaire (MQ) [59]. The scale consists of 21 items assessing fatigue, difficulties falling asleep, apathy, irritability, energy loss, depression, and waking up exhausted. A sample item is “Do you sometimes feel as if your body is like a battery that is losing its power”? Each of the 21 items can be rated on a three-point scale, from “statement is not true” (1) to “undecided” (2) or “true” (3). By summing the scores of each item, the total score could be calculated, with higher scores indicating increased vital exhaustion. Reliability was α = 0.88.

#### 2.2.2. Classroom Observations

GoPro cameras and microphones were installed in each classroom to assess four consecutive, same-day lectures per teacher. After being trained to a criterion of 0.80 agreement (Cohen’s kappa), observers coded student aggression using MAXQDA Analytics Pro 2020 version 20.4.1 in an event sampling procedure using the observation system BASYS [14]. For the present study, 19 interactional episodes with a high density of aggression were subjected to microanalysis. These episodes were fully transcribed considering verbal, paraverbal, and nonverbal aspects according to the transcription rules of Fuss and Karbach [69]. Subsequently, student and teacher behavior was coded using the BAVIS [13] and BASYS [14] observation systems.

Student behavior included three overarching categories: aggressive (insulting, hitting, and spreading false rumors), disruptive (behavior that interferes with lessons but is not aggressive), and pro-social (behavior appropriate to the teaching standards). Teacher behavior included three overarching categories: punitive (threatening, punishing, and belittling), neutral (observing/ignoring, stopping, and admonishing), and social integrative (suggesting compromise, integrating, encouraging, empathizing, and using humor). The SSG method [15] was used to represent reciprocally related student and teacher behaviors in state space grids. The coded data were exported from MAXQDA Analytics Pro 2020 version 20.4.1 and processed in Excel (version 2402) to create the state space grids. A two-dimensional system graphically represented interactions between the teacher and the class. In the present study, the teacher’s behavior formed the y-axis, and the student’s behavior formed the x-axis, resulting in one SSG for each interactional episode. A category system with three categories on each axis resulted in an SSG with nine cells, representing all possible combinations of behaviors of both parties.

#### 2.2.3. Heart Rate Variability

The teachers’ HR and HRV were measured continuously using an ambulatory electrocardiogram (ECG). HRV measurement is sensitive to methodological aspects [70]. For the assessment of HRV, we followed the guidelines of the task force on HRV [47]. ECG data were captured with the EcgMove3 sensor from Movisens Version 1.9.31.0 (movisens GmbH, Karlsruhe, Germany), which is easy to use. The sensor records a single-channel ECG at a sampling rate of 1024 Hz. The teachers attached adhesive electrodes to the sensor upon awakening, and the ECG data were collected continuously until 8:00 pm. The sensor also measures physical activity based on the registration of acceleration in three dimensions and atmospheric air pressure. For ECG data processing and analysis, Kubios HRV Premium version 3.0.2 was used [71]. The raw data were edited manually in Kubios. After artifact correction, the data were exported into SPSS. HRV was assessed during aggression and control episodes. Each control segment had the same duration as the corresponding aggression segment (range 30–169 s; *M* = 57 s). Control episodes were timed at least 3 min ahead, had the same duration, had no higher level of classroom disruption, were in the same setting, and had no strong teacher movements or aggression 5 min before or during the episode. We relied on two HRV parameters that are often used in research [72]: RMSSD (root mean square of successive differences between normal heartbeats) and SDNN (standard deviation of the NN interval).

RMSSD corresponds to the beat-to-beat variance in heart rate and is considered the standard measure of parasympathetic (vagally) mediated cardiac regulation [72]. SDNN expresses the overall variability of the temporal fluctuations of the heartbeat. The SDNN indicates the autonomic nervous system’s regulatory capacity and the cardiovascular system’s adaptability [72].

#### 2.2.4. Progesterone

Progesterone was determined from a hair sample collected from the posterior vertex region of the head while using the 3 cm segment closest to the scalp. Given an average hair growth of 1 cm per month, this segment represents the cumulative glucocorticoid secretion over three months before sampling [73]. Progesterone was measured using liquid chromatography–tandem mass spectrometry (LC–MS/MS) [74].

#### 2.2.5. Overweight (BMI)

Weight and height were recorded to determine BMI [75]. BMI was calculated by dividing the teachers’ weight [kg] (Seca 813; Reinach, Switzerland) by the square of their height [m] (Seca 213; Reinach, Switzerland).

### 2.3. Data Analyses

All the data were analyzed using IBM SPSS Statistics (Version 29). Normal distribution was tested using the Shapiro–Wilk test, and skewed variables were log-transformed using the natural logarithm (ln(x + 1)). Potential confounders (i.e., sex, age, and BMI) were tested for significant influences on the main variables (i.e., stress, resources, and risk variables) in bivariate correlations.

Research questions 1 and 2 were examined with the SSG method. Due to the small sample size, research question 3 was not examined correlatively but only exploratively by comparing group averages. For research question 4, we subtracted the mean RMSSD, mean SDNN, and mean HR of the aggression episode from the mean RMSSD, mean SDNN, and mean HR of the control episode for each teacher, resulting in three difference variables (RMSSD_d, SDNN_d, and HR_d). For research question 5, these differences were then correlated with bivariate Pearson correlations with resources and risk factors as well as stress outcomes. The significance level was set at *p* < 0.05.

## 3. Results

The teachers in the present subsample did not differ significantly from those in the overall sample in terms of all variables reported here. A Mann–Whitney U test showed more observed student aggression in the present subsample (*Mdn* = 1.25) than in the overall sample (*Mdn* = 0, U = 18.00, asymptotic *p* < 0.001, *r* = 0.72).

### 3.1. Student Aggression and Teacher Responses

Teacher responses to student aggression and teacher behavior in overall interactional turns are presented in Table 1.

*Interaction density.* The 19 examined episodes were characterized by a very high interactional density, with an average of over 15 speaker turns per minute. A speaker change occurred every four seconds. We observed 258 student responses (11.73 per minute) and 150 teacher responses (6.82 per minute). The teachers were exposed to separable student behaviors every 5 s and responded to these behaviors every 8 to 9 s.

*Aggressive student behavior.* In the examined episodes, there were 167 incidents of student aggression (7.59 per minute). We observed physical aggression against other students in 84 cases (50.3%), verbal aggression against students in 50 cases (29.9%), aggressive–oppositional behavior against the teacher in 20 cases (12.0%), and physical aggression against objects in 13 cases (7.8%).

*Teacher responses to student aggression.* In 89.8% of the cases, teachers reacted neutrally to aggressive student behavior, with the most frequent subcategory being ignoring (65.9%). In 6.0% of the cases, they reacted punitively, and in 2.4% of the cases, they reacted in a socially integrative manner.

*Teacher behavior in all interactional turns:* The most frequent teacher behavior in all interaction terms was neutral (80.9%), followed by punitive (11.4%) and social integrative behavior (3.7%). The admonishing behavior category included three subcategories: Most often, teachers ignored student behavior (53.5%), admonished students (14.1%), or stopped the behavior (13.4%).

*Aggressive teacher behavior*. In 20 cases (0.91 per minute), teachers showed aggressive behavior toward students, such as by belittling them through ironic criticism or embarrassment.

### 3.2. Interactional Episodes

A student’s aggression and the subsequent teacher response can develop into longer interactional episodes in which students and teachers react to each other’s behaviors. Table 2 shows that by definition, the overall system of dyadic social interactions under study (teachers and students) can assume nine possible states defined by the combination of reciprocally interrelated teacher and student behaviors.

By far, the most frequent type of interaction, with a total of 50.2%, consisted of a combination of neutral (mostly ignoring) teacher behavior and aggressive student behavior, followed by a combination of neutral (mostly ignoring) teacher behavior and disruptive (18.1%) and prosocial (11.4%) student behaviors.

In the following, we present two examples: Since ignoring does not interrupt aggressive or disruptive student behavior, the interactional system remains in the unfavorable state of ignoring teacher behavior and continuing aggressive student behavior, and the interactional system remains in the same unfavorable state over several interactional turns (see example 1).

Example 1. *Start of class: Several students fight with each other and throw each other to the ground. Most of the students are watching them. The teacher tries to stop the aggressive behavior for the time being with two timid termination attempts (Hei!) and ignores the following physical aggression of the students. The interactional system remains in the state of the teacher ignoring the aggressive behavior for over 2 min and 48 s, and the students show 23 instances of physical aggression (8.2 per minute). (Teacher 30, transcript 1, lines 1–134)*

Teachers responded in a socially integrative manner to aggressive student behavior only 1.3% of the time (see example 2).

Example 2. *The work atmosphere is unfocused. One student yells into the classroom. Another grabs the bracket for the blinds and swings it back and forth; one student pounds the air with his fist, and another refuses the assigned task. The teacher asks, “Do you want us to hold still for a second, or can you do it”? Students look at the teacher silently and then answer, “Yes”. The teacher can continue the lesson undisturbed. (Teacher 2, transcript 2, lines 68–106)*

However, teachers differed in terms of the variability of their behavior. Two teachers showed high variation and responded flexibly to challenging student behavior, while eight teachers reacted rigidly and showed hardly any variation in their behavior.

### 3.3. Teacher Responses in the Light of Resources and Risk Factors

The overarching question here was, “Which factors promote an adaptive teacher response to aggressive student behavior?” In the following, we analyzed the context of aggressive student behaviors using the variables described above (Table 3). Interactional turns and student aggression were assessed per minute.

Due to the small sample size, we could not calculate the statistical significance of differences between the kinds of teacher responses to student aggression. However, a few descriptive trends emerged. Teachers who showed punitive teacher responses in aggressive interactional episodes had slightly higher mean values for pressure to succeed, work overload, and occupational problems than the other groups. In contrast, teachers who showed social integrative responses had higher mean values for work satisfaction and progesterone and lower mean values for work overload. We found comparable patterns in bivariate correlations. However, the correlations are not reported here due to the small sample size and heterogeneity of the effects of risk factors and resources.

### 3.4. Teachers HRV in Aggressive Interactional Episodes and Associations with Risk Factors and Resources

The first question we examined was, “How do teachers respond physiologically to aggressive student behavior?” In 15 of 20 cases (75%), teachers responded with a pattern of downregulation (defined as a higher HRV and a lower HR during the aggression than during the control episode). In contrast, in 5 of 20 cases (25%), teachers responded with a pattern of upregulation (a lower HRV and a higher HR during the aggression than during the control episode).

Subsequently, we examined the question, “How are teachers’ resources and risk factors associated cross-sectionally (t0) with teachers’ up and downregulation?” The protective factors seeking positive experiences and support from school administration were associated with more downregulation and less upregulation of the teacher when student aggression occurred. Specifically, Table 4 shows that seeking positive experiences was associated with a lower HR and social support with a higher HRV in aggressive interactional episodes. In contrast, progesterone was not significantly associated with HR and HRV.

The risk factors of self-related problems, vital exhaustion, work overload, and an elevated BMI were associated with more teacher upregulation and less downregulation. Specifically, work overload and vital exhaustion were associated with a higher HR. Self-related problems were associated with a reduced SDNN and higher HR, whereas no significant associations were found for occupational problems (see Table 4).

### 3.5. Long-Term Effects of Upregulation

Finally, we tried to answer the question, “How are up and downregulation longitudinally associated with teachers’ psychological strain?” Table 4 presents that longitudinally, higher work overload, self-related problems, vital exhaustion, and BMI were partially associated with more upregulation and less downregulation. However, the effects differed for the individual indicators. A reduced RMSSD was longitudinally associated with an increased BMI. A reduced SDNN was associated with self-related problems at t0 and with BMI after two years. Further correlations pointed in the expected direction but did not reach significance. The effects were clearest for HR. More upregulation and less downregulation in HR were consistently associated with increased work overload, self-related problems, and vital exhaustion across all measurement times. For occupational problems and BMI, the effects were only partially consistent.

## 4. Discussion

The main aim of the present study was to investigate teachers’ physiological and behavioral responses when facing aggressive student behavior and to examine which resources foster adaptive teacher reactions. We found that teachers mostly ignore aggressive student behavior, potentially contributing to prolonged episodes of aggressive behavior over several interaction turns. The resources of high progesterone, high work satisfaction, and low levels of work overload favored social integrative teacher responses. In contrast, the risk factors pressure to succeed, work overload, and occupational problems were associated with punitive teacher responses. In 75% of cases, teachers succeeded in downregulating their physiological activity (increase in HRV, decrease in HR) in aggressive interactional episodes. Assessed after two years, teachers’ capacity to downregulate their physiological stress reaction in aggressive interactional episodes seems to have protected teachers at least partially from long-term psychological strain.

### 4.1. Student Aggression and Teacher Reaction

Hypothesis 1, assuming that teachers mostly ignore aggressive student behavior, was confirmed. Our explorative study shows that teachers face an enormous density of heterogeneous interactions. Many things happen simultaneously, and teachers need to be aware of the different social processes in the classroom and make quick decisions. The complexity of social situations in the classroom can lead to excessive demands and may also explain why teachers often ignore aggressive student behavior as a way of coping. This finding aligns with two extensive observational studies on aggressive behavior in schools [13,14] showing that teachers ignore aggressive student behavior 75% to 95% of the time. However, ignoring aggressive student behavior may become problematic over time because students might interpret the lack of teachers’ response as approval and might not stop the behavior. That we observed a low frequency of social integrative teacher reactions to aggressive student behavior is an unfavorable finding, as studies show that encouragement, empathy, and humor are highly effective strategies for successfully stopping aggressive student behavior without damaging the relationship [14].

Teachers sometimes react aggressively to students by belittling them through ironic criticism or embarrassment. Although many aggressive teacher responses might be short-term responses to stress rather than planned, deliberate actions, they still have far-reaching consequences. Teacher aggression does not only affect the victims (i.e., the students). It can also have an impact on witnesses. Indirect exposure to teacher aggression can damage the learning process by distracting from the task [76,77]. Research has generally shown that aggressive teacher behaviors affect the target and undermine the behavioral engagement of bystanders [78] as well as the teacher–student relationship [4].

### 4.2. Interactional Episodes

Hypothesis 2, expecting that the most frequent combination of observed behaviors is aggressive student behavior and ignoring teacher behavior, was confirmed. Teachers often ignore aggressive student behavior, which appears to contribute to repeated aggressive student behavior over several interactions. Prolongation of aggressive episodes appears to be unhealthy for both the teacher and the involved students, undermining teaching-and-learning processes and, consequently, the student’s learning outcomes. Several reasons can explain the lack of teacher response. First, studies have shown that teachers are not consciously aware of all the aggression in the classroom [14]. Second, even if an incidence of student aggression is perceived, a teacher may consciously decide not to react in order to not interrupt the lesson flow. Third, it is conceivable that the teacher is overwhelmed and feels that personal resources are too low to stop the aggressive behavior. Fourthly, it is also possible that some teachers habitually have a strong need for harmony and are afraid of being disliked by the students if they intervene.

The low variability of teacher behavior during aggressive interactional episodes suggests that teachers respond relatively uniformly and automatically to student behavior. Automatic responses probably indicate a lack of conscious decision-making and a lack of consideration of alternative actions in stressful situations. Instead, teachers respond relatively automatically and stereotypically to aggressive student behavior. Thus, they unintentionally limit their pedagogical scope and do not respond adaptively to challenging student behaviors. Teacher education will benefit from supporting teachers in questioning their habitual response patterns, reacting early and at a low level of interactional tension to problematic student behavior, and preventing the exacerbation of aggressive interaction chains. The teacher’s presence and socially integrative measures are suitable for this purpose.

In contrast, punitive and moralizing behavior on the part of the teacher has been shown to be counterproductive, as it exacerbates the problem [79]. It is particularly striking that teachers rarely respond in a socially integrative manner. A well-known study of teacher behavior in aggressive interactional episodes [80] clearly showed that although teachers recommend social integrative measures to novice teachers, they hardly implement them in their own teaching. The great divergence between recommendations and actions in everyday school life raises the question of whether teachers “in the eye of the storm” of student aggression are detached from educational and longer-term objectives in handling student aggression so that this advice represents rather abstract concepts or wishful thinking [80].

### 4.3. Teacher Reaction in the Context of Resources and Risk Factors

Hypothesis 3 proposed that teachers’ resources foster social integrative teacher reactions, whereas risk factors are associated with punitive teacher reactions. The hypothesis was partially supported. Our study suggests that the potential resources progesterone, work satisfaction, and low levels of work overload support social integrative teacher responses. In contrast, the risk factors of pressure to succeed, work overload, and occupational problems are associated with punitive teacher responses. Teachers are assumed to evaluate stressors in light of their resources [27]. When they perceive their resources as insufficient to cope with the challenging situation, stress arises, and subsequently, they react inefficiently to aggressive behavior. This aligns with Conservation of Resources Theory [81], that posits that stress emerges from situations involving threatened loss of valued resources. It is, therefore, crucial to strengthen the teachers’ coping resources wherever possible. While we cannot influence teachers’ progesterone levels in pedagogical practice, reducing the pressure to succeed and work overload offers important starting points for preventing unfavorable teacher reactions to aggressive student behavior.

### 4.4. Teachers’ HRV in Aggressive Interactional Episodes

Aggressive student behavior is considered one of the primary stressors for teachers. Therefore, one might assume sympathetic physiological activity consistently increases when student aggression occurs, resulting in decreased HRV and increased HR. However, our analysis showed that this *fight or flight pattern* (upregulation, defined as a lower HRV and a higher HR during the aggressive episode than during the control episode) occurs in only 25% of the cases. In 75% of the cases, teachers downregulate their physiological activity in this stress-inducing situation (defined as a higher HRV and a lower HR during the aggression than during the control episode): their polyvagal brake kicks in, their HR decreases, and their HRV increases. In evolutionary terms, this polyvagal brake is seen as a prerequisite for living together in complex social groups. The school class is a prototype of such a social system. Therefore, it is very fortunate that, in most cases, teachers managed to regulate themselves physiologically and did not react aggressively to aggressive student behavior.

Hypothesis 4—that teacher resources promote downregulation, whereas risks and psychological strain are more likely to foster mobilization—was partially supported. We have seen that seeking positive experiences and receiving support from school administration are positively associated with a teacher’s downregulation when student aggression occurs. In contrast, progesterone was not significantly associated with teachers’ HR and HRV. Seeking positive experiences is crucial for recovery. A study by Virtanen and colleagues [82] showed that recovery is associated with emotion regulation. Recovery buffers the effects of high job demands and is crucial for teachers’ well-being. Consequently, we need to support teachers in fostering their recovery skills by building personal resources through meaningful leisure activities that help teachers cope with upcoming stress. Thus, a way to protect teachers from unfavorable physiological stress reactions is to encourage them to seek positive experiences outside of work and to create a supportive school environment, especially regarding support by the school administration.

### 4.5. Long-Term Effects of Upregulation

Hypothesis 5, that upregulation is longitudinally associated with teachers’ psychological strain, was partially confirmed. There are clear differences between the various indicators of upregulation. While the harmful effects of upregulation are most evident in HR, the associations are less strong and less consistent for RMSSD and SDNN, but they point in the expected direction. Thus, over two years, teachers’ capacity to downregulate their physiological stress response in aggressive interactional episodes is associated with more favorable outcomes in terms of psychological strain than it is for teachers who upregulate. Thus, the polyvagal brake seems to successfully downregulate the sympathetic nervous system [54] and seems to longitudinally protect teachers at least partially from psychological strain.

Undoubtedly, teaching is demanding, and teachers rely on support from their school teams to fulfill their challenging tasks. Our study shows that perceived support positively influences teachers’ cardiac reactions when facing student aggression. This result also aligns with a finding using a larger sample of the present study [36], which shows that perceived support from other teachers and the school administration prevents unfavorable physiological stress consequences such as high BMI, blood pressure, and hair cortisol. At the same time, however, this finding also shows that previous stress experiences unfavorably affect teachers’ acute physiological stress reactions. This aligns with more recent stress models [36,63] that emphasize the cumulative effects of stress exposure and its influence on acute stress responses.

### 4.6. Strengths, Limitations, and Implications

This ambulatory assessment study holds several strengths. It goes far beyond analyses of questionnaire data alone by examining teachers’ moment-to-moment interpersonal behavior and cardiovascular activity in the light of polyvagal theory. However, this exploratory study is limited by a small sample of apparently healthy teachers and does not allow generalized statements about the extent of aggressive episodes in schools. Beyond that, this study focused on teachers in Switzerland. However, teachers’ responses to aggressive student behavior may vary culturally. Thus, generalizing the results to teachers in different countries requires caution. Furthermore, in this study, female teachers are overrepresented (90%) compared to the overall sample (66.7%). However, student aggression was not associated with teachers’ gender, years of teaching experience, or school level in the overall sample. Women might choose to work in hot-spot classes (i.e., classes with an above-average number of challenging students with behavioral problems) more often than men. In addition, aggressive student behavior was not very frequent in the overall study. We attribute this to a possible reactivity of teachers and students. The recordings by cameras and microphones, teachers wearing an ECG, and the frequent provision of salivary saliva samples (method/data not presented here; see Schneider et al. [83]) led most likely to a repeated or even constant awareness of being observed, which might undermine typical habituation effects.

Although we could confirm some of our study hypotheses, others were confirmed only partially. In hypothesis 4, for example, the relation of seeking positive experiences with punitive and socially integrative teacher reactions went in an unexpected direction. Regarding hypothesis 5, not all resources seemed to have an equally strong relationship with teachers’ cardiac parameters. However, the small size of our subsample limited the possibilities of statistical testing, which might contribute to such inconclusive results. Thus, further studies with a larger sample are needed to understand these phenomena. Finally, in future research, the sequential order of aggressive student behavior followed by teacher responses could also be thought of in the opposite direction.

The present study offers several practical implications. Recovery has a pivotal role in stress and emotion regulation [82]. In teacher education or training courses, teachers should be made aware of suitable coping strategies such as building personal relationships and seeking support. Thus, teachers should actively learn to shape their recovery adaptively, counteracting stress and, consequently, maladaptive teacher reactions. Further, school administrators are valuable for stress prevention among teaching staff through social support. It is important to note that school administrators might underestimate their role in supporting teachers and preventing stress among the teaching staff. Understanding and acknowledging this responsibility is the first step toward effective action.

## 5. Conclusions

This study used a psychophysiological ambulatory assessment approach to examine teachers’ behavior and stress response in aggressive teacher–student interactions. Aggressive student behavior is a crucial stressor for teachers, and teachers may be at risk of aggravating the challenging interactional situation through their maladaptive coping strategies. Progesterone, work satisfaction, and low levels of work overload seem to be crucial resources that favor social integrative teacher responses. Over two years, teachers’ ability to physiologically downregulate in aggressive interactional episodes seems to longitudinally protect them from adverse longitudinal consequences of psychological stress, whereas physiological upregulation appears to be associated with elevated psychological strain. Thus, teacher education appears well-advised to strengthen the teacher’s coping resources and strategies regarding aggressive student behavior. Finally, perceived support from other teachers and the school administration is crucial in preventing unfavorable physiological stress consequences in teachers.

## Figures and Tables

**Table 1:** Teacher responses to student aggression and teacher behavior in overall interaction turns.

	Teacher Responses to Student Aggression		Teacher Behavior in Overall Interaction	
	*n*	%	*n*	%
Social integrative	4	2.4	11	3.7
Humor	0	0	0	0
Empathize	1	0.6	4	1.3
Encourage	1	0.6	4	1.3
Integrate	2	1.2	2	0.7
Suggest compromise	0	0	1	0.3
Neutral	150	89.8	242	80.9
Observe/ignore	110	65.9	160	53.5
Stop	17	10.2	40	13.4
Admonish	23	13.8	42	14.1
Punitive	10	6.0	34	11.4
Threaten	1	0.6	8	2.7
Punish	2	1.2	6	2.0
Belittle	7	4.2	20	6.7
Other	3	1.8	12	4.0
Total	167	100.0	299	100.0

**Table 2:** Interaction patterns in aggressive episodes over all classes and teachers.

**Teacher behavior**	social integrative	1.3%	0%	2.0%
	neutral	50.2%	18.1%	11.4%
	punitive	3.3%	4.0%	3.3%
		aggressive	disruptive	prosocial
		**Student behavior**		

Note. The full 100% refers to 299 turns. The values are percentages. In 6.4% of the cases, the teacher’s or the student’s behavior could not be assigned to any observational category, and the category “other” was assigned.

**Table 3:** Means of study variables split into punitive, non-punitive, social, and non-social responses of teachers.

Variable	Punitive	Non-Punitive	Social	Non-Social
1. Interaction turns ^a^	15.25	14.00	15.67	14.00
2. Student aggression ^a^	7.65	6.05	7.12	6.51
3. Seeking positive experiences	3.88	3.75	3.50	3.93
4. Support from school team	4.50	4.67	4.00	4.86
5. Support from school administration	3.00	3.83	3.00	3.71
6. Work satisfaction	4.65	4.83	5.07	4.63
7. Progesterone	1.10	1.24	1.83	0.91
8. Pressure to succeed	1.83	1.43	1.44	1.65
9. Work overload t0	1.65	1.50	1.37	1.64
10. Occupational problems t0	2.13	1.63	1.77	1.86
11. Self-related problems t0	1.75	1.83	1.53	1.91
12. Vital exhaustion t0	1.44	1.53	1.37	1.55
13. Body mass index t0	24.28	22.79	23.17	23.48

*Note.* ^a^ per minute, *N* = 10, punitive *n* = 4, non-punitive *n* = 6, social *n* = 3, non-social *n* = 7.

**Table 4:** Correlations between study variables and differences in aggression and control episodes for RMSSD, SDNN, and HR.

Variable	RMSSD_d	SDNN_d	HR_d
Seeking positive experiences	−0.03	−0.05	−0.70 *
Support school administration	−0.77 **	−0.85 **	−0.09
Progesterone	0.23	−0.02	−0.06
Work overload t0	0.06	0.29	0.74 **
Work overload t1	0.19	0.72 *	0.89 **
Work overload t2	0.22	0.07	0.72 *
Occupational problems t0	0.17	0.52	0.30
Occupational problems t1	−0.03	0.40	0.74 *
Occupational problems t2	0.23	−0.11	0.59
Self-related problems t0	0.44	0.67 *	0.74 **
Self-related problems t1	0.36	0.46	0.89 **
Self-related problems t2	0.12	0.03	0.70 *
Vital exhaustion t0	0.19	0.52	0.63 *
Vital exhaustion t1	−0.04	0.31	0.72 *
Vital exhaustion t2	−0.26	0.02	0.58
BMI t0	0.82 **	0.51	0.08
BMI t1	0.70 *	0.22	0.41
BMI t2	0.76 *	0.63 *	0.75 *

*Note. N* = 10, t1 and t2 *n* = 8, RMSSD = root mean square of successive differences, SDNN = standard deviation of the NN interval, HR = heart rate, d = difference between control minus aggression episode for RMSSD and SDNN or difference between aggression minus control episode for HR, BMI = body mass index; positive correlations: more upregulation/less downregulation, negative correlations: more downregulation/less upregulation; * *p* < 0.05, ** *p* < 0.01 (one-tailed).

## Data Availability

The data presented in this study are available upon request from the corresponding author. The data are not publicly available due to privacy concerns.
